# Atrioventricular Dissociation after Electroconvulsive Therapy

**DOI:** 10.4061/2011/746373

**Published:** 2011-09-15

**Authors:** Siegfried William Yu, Srikanth Vallurupalli, Jennifer Arnoldi, Richard Holloway

**Affiliations:** ^1^Department of Internal Medicine, Southern Illinois University School of Medicine, Springfield, IL 62711, USA; ^2^School of Pharmacology, Southern Illinois University Edwardsville and St. John's Hospital, Springfield, IL 62711, USA; ^3^Southern Illinois University School of Medicine and Prairie Cardiovascular Consultants, Springfield, IL, USA

## Abstract

Electroconvulsive therapy (ECT) is increasingly used as a treatment for psychiatric disorders. Cardiac effects are the principal cause of medical complications in these patients. We report a case of atrioventricular (AV) dissociation that occurred after ECT that was treated with pacemaker implantation. The mechanisms contributing to the onset of AV dissociation in this patient, and the management and rationale for device therapy, in light of the most recent guidelines, are reviewed.

## 1. Introduction

Electroconvulsive therapy (ECT) has been used as a treatment for psychiatric disorders since the 1930s. It is a very useful treatment for severe depression that is being used increasingly [1] and is considered the most effective treatment in psychiatry [2]. A number of large studies and meta-analyses confirm the efficacy and safety of ECT. Cardiac complications are the principal cause of medical complications related to ECT [3]. Although they are usually insignificant, a small percentage of these cardiac complications can be potentially fatal. We report a case of complete atrioventricular (AV) block that occurred after ECT, discuss the possible mechanisms involved, and review the management of the case.

## 2. Case Report

**Figure 1:**
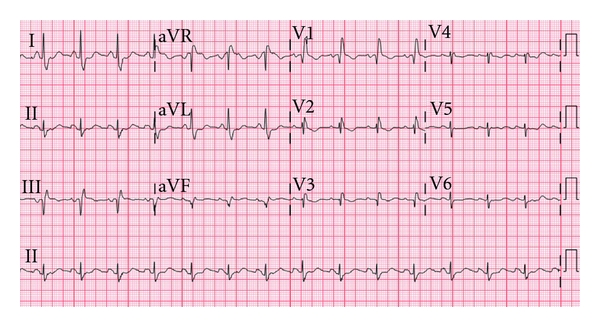
Patient's baseline electrocardiogram.

A 65-year-old woman with type 1 bipolar disorder, hypothyroidism, hypertension, and hyperlipidemia was admitted to the psychiatric unit with worsening depression and inability to care for self at home. She had no documented history of coronary artery disease or previous myocardial infarction. Her medications included risperidone, venlafaxine, bupropion, aspirin, levothyroxine, simvastatin, lorazepam, and gabapentin. Upon admission bupropion and gabapentin were discontinued, and escitalopram and mirtazapine were added. Because her depression was resistant to medical therapy, ECT was pursued. Her baseline ECG (see Figure 1) demonstrated sinus rhythm, with a right bundle branch block (RBBB) pattern and Q waves in leads III and aVF suggesting a previous inferior infarct. The PR interval was normal at 160 milliseconds (ms). Hydrochlorothiazide and amlodipine were prescribed for her untreated hypertension.

**Figure 2:**
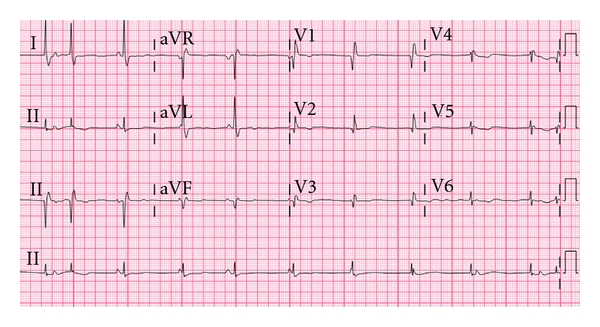
AV dissociation occurring after the 8th ECT session.

She was started on three sessions of unilateral ECT a week, with a frequency of 60 hertz and a current of 800 milliamperes. Premedication for each ECT session consisted of intravenous methohexital and succinylcholine. Intravenous labetalol was given as needed before and after the ECT sessions for elevated blood pressure. She tolerated the initial sessions well. Prior to her 5th ECT session, she was started on oral metoprolol succinate at a dose of 25 mg daily for uncontrolled blood pressure. Her blood pressure remained elevated, and the dose was increased to 50 mg daily prior to her 8th ECT session. The seizure duration during her 8th ECT session was 238 seconds, which did not differ significantly from the prior sessions (range 20–458 seconds). Thirty minutes after her 8th ECT session, the patient was noted to be bradycardic in the post-ECT monitoring area. The patient was asymptomatic with a blood pressure of 120/80 mm Hg. A 12-lead ECG (see Figure 2) revealed an atrial rate of 52 per minute and a wide QRS ventricular rate of 60 per minute, consistent with AV dissociation, with a QRS morphology resembling her preexisting RBBB and a QTc interval of 416 ms. 

She was transferred to the coronary care unit, where the rhythm persisted for approximately 6 hours before a repeat 12-lead ECG confirmed return to sinus rhythm. Her thyroid-stimulating hormone levels were within normal limits. A transthoracic echocardiogram revealed severe left ventricular (LV) hypertrophy, and an ejection fraction of 80% with near obliteration of the left ventricular cavity on systole, creating an LV outflow tract obstruction not affected by valsalva maneuver. The LV diastolic filling pattern was consistent with impaired LV relaxation. Electrophysiological consultation was obtained, and she underwent implantation of a dual-chamber pacemaker. Regadenoson radionuclide stress imaging was negative for cardiac ischemia. She underwent 3 subsequent ECT sessions without complications. Her mood progressively improved, and she was eventually discharged home.

## 3. Discussion

The Global Burden of Disease Study projects that, by 2020, depression will be second only to ischemic heart disease worldwide as a leading cause of disability-adjusted life years [4]. Due to this expected increase in prevalence for both ischemic heart disease and depression and the increasingly widespread use of ECT, it is essential for physicians to recognize and manage ECT-related cardiac complications—the most common adverse effect of ECT. 

The variety of cardiac complications of ECT may be grouped in terms of cardiac ischemia, heart failure, and arrhythmic complications. Ischemic complications reported include both ST segment and non-ST segment elevation myocardial infarction [5]. Severe congestive heart failure resulting from new-onset atrial fibrillation may occur [6]. Arrhythmic complications are by far the most common. They include minor transient arrhythmias, such as premature ventricular contractions, but also significant tachyarrhythmias and bradyarrhythmias, such as atrial flutter, atrial fibrillation, supraventricular tachycardia, ventricular tachycardia, new left and right BBBs, 2nd degree AV block, junctional bradycardia, and asystolic cardiac arrest [5–7]. Routine preoperative evaluation includes a careful history and physical examination. In the absence of active cardiac disease (heart failure, arrhythmia, or angina), the only test recommended is an ECG especially in patients above 50 years of age [8].

AV dissociation and complete AV block complicating ECT has been rarely reported. In 1972, Malik reported complete AV block in a 33-year-old female without any known medical problems, or any other concurrent medications, after her 1st ECT session [9]. This patient received premedication with methohexital, succinylcholine, and atropine. Twenty minutes after the procedure, she was found to be apneic and in pulseless cardiac arrest. She was resuscitated, and her ECG revealed complete AV block. Despite emergent transvenous pacemaker implantation, the patient deteriorated into ventricular fibrillation and expired.

The arrhythmic complications of ECT can be easily understood through the concept of seizure-induced autonomic nervous system activity. During the early phase of the seizure, parasympathetic activity predominates with a fall in pulse rate and blood pressure. This is followed by a sympathetically induced rise in pulse rate and blood pressure. These physiological responses can precipitate a variety of cardiac complications especially in patients with preexisting heart disease [7]. Bradyarrhythmias are likely to occur when the parasympathetic activity predominates while tachyarrhythmias are a consequence of unopposed sympathetic activity.

During ECT, periprocedural anesthetics are used to produce loss of consciousness with minimal convulsive motor activity. Short-acting agents are generally preferred given the short duration of seizure activity and the ability to medicate without intubation. Methohexital for induction of anesthesia and succinylcholine for neuromuscular blockade are considered drugs of choice [10]. Both of these agents were used in our patient and are not known to cause AV conduction delay. 

Anticholinergic therapy with atropine or glycopyrrolate may be useful in preventing severe bradycardia associated with the initial parasympathetic response, especially for patients with pre-existing bradycardia or AV block. There is a difference of expert opinion regarding the routine use of anticholinergic agents with ECT [11, 12]. The use of these medications may not prevent bradyarrhythmias, as shown in the case reported by Malik, where complete AV block occurred despite anticholinergic therapy [9].

Beta-blockers such as labetalol and esmolol are commonly used to control sympathetically induced tachycardia and hypertension associated with ECT. Labetalol, a combined alpha and beta-adrenergic antagonist, is effective within 5 minutes of administration with a half-life of 4–6 hours. Esmolol is a selective beta-adrenergic antagonist that is effective within 30–90 seconds but has little sustained activity after 10 minutes. Although esmolol is often considered the drug of choice in ECT due to its favorable pharmacokinetic profile, labetalol continues to be widely used [10].

Drug interactions between psychotropic and cardiac medications may also predispose to bradyarrhythmias. Venlafaxine and escitalopram, which were used in our patient, both inhibit the 2D6 isoenzyme of the cytochrome P450 system, thereby decreasing the metabolism of, and resulting in increased concentrations of metoprolol [13].

The mechanism of AV dissociation in our patient was multifactorial. The introduction of metoprolol succinate and more importantly dose escalation before the 8th ECT session, on a background of structural heart disease and pre-existing conduction abnormality (RBBB), was her first risk factor for inhibition of AV conduction. Amlodipine, which was also used, has no significant electrophysiological action on sinus or AV node function in patients receiving beta-blockers [14]. Psychotropic drug interactions, however, heightened the intensity of beta-blockade. During ECT, the initial post-ECT parasympathetic surge combined with the administration of labetalol worsened AV conduction and provoked AV dissociation. 

AV block due to medication often resolves with the discontinuation of the offending agent but frequently recurs, as illustrated by Zeltser et al. [15]. In their study, 41% of patients (who were receiving beta-blockers and/or calcium-channel blockers) converted to sinus rhythm within 48 hours after discontinuing the medication. However, 56% of these patients had recurrence of AV block within the 3 weeks following discontinuation. Zeltser et al concluded that while AV block is commonly “related to drugs,” it is rarely “caused by drugs” and noted that these patients frequently still require pacemaker implantation. In such cases, the 2008 Guidelines for Device-Based Therapy of Cardiac Rhythm Abnormalities from the American College of Cardiology leave this decision to the purview of the treating physician [16]. They designate a Class I indication for permanent pacemaker implantation in asymptomatic persistent third-degree AV block at any anatomic site with average awake ventricular rates of 40 beats per minute or faster if cardiomegaly or LV dysfunction is present, or if the site of block is below the AV node.

In our patient, continued beta blocker therapy was necessary to control her hypertension which was not responsive to two other medications and as therapy for her structural heart disease. Further sessions of ECT were needed to treat her depression. The use of intravenous beta-blockers to blunt the sympathetic surge associated with ECT would have placed her in the same milieu that had provoked her episode of AV dissociation. Pacemaker implantation was thus justified based on these indications. The patient received appropriate preoperative therapy of her hypertension.

## 4. Conclusion

In summary, we describe the occurrence of AV dissociation as a complication of ECT. The mechanism involved patient-related, pharmacologically predisposed, and ECT-related factors. This case highlights the occurrence of cardiac bradyarrhythmias with ECT and the need for physicians to maintain vigilance in detecting and treating these complications, in order to prevent potentially fatal patient outcomes.
